# Dynamic interspecies interactions and robustness in a four‐species model biofilm

**DOI:** 10.1002/mbo3.1254

**Published:** 2021-12-07

**Authors:** Aurélie Baliarda, Michèle Winkler, Laurent Tournier, Colin R. Tinsley, Stéphane Aymerich

**Affiliations:** ^1^ INRAE, AgroParisTech, Micalis Institute Université Paris‐Saclay Jouy‐en‐Josas France; ^2^ INRAE, MaIAGE Université Paris‐Saclay Jouy‐en‐Josas France

**Keywords:** adhesion, biofilm, microbial ecology, microbial interactions and pathogenesis

## Abstract

Interspecific interactions within biofilms determine relative species abundance, growth dynamics, community resilience, and success or failure of invasion by an extraneous organism. However, deciphering interspecific interactions and assessing their contribution to biofilm properties and function remain a challenge. Here, we describe the constitution of a model biofilm composed of four bacterial species belonging to four different genera (*Rhodocyclus* sp., *Pseudomonas fluorescens, Kocuria varians*, and *Bacillus cereus*), derived from a biofilm isolated from an industrial milk pasteurization unit. We demonstrate that the growth dynamics and equilibrium composition of this biofilm are highly reproducible. Based on its equilibrium composition, we show that the establishment of this four‐species biofilm is highly robust against initial, transient perturbations but less so towards continuous perturbations. By comparing biofilms formed from different numbers and combinations of the constituent species and by fitting a growth model to the experimental data, we reveal a network of dynamic, positive, and negative interactions that determine the final composition of the biofilm. Furthermore, we reveal that the molecular determinant of one negative interaction is the thiocillin I synthesized by the *B. cereus* strain, and demonstrate its importance for species distribution and its impact on robustness by mutational analysis of the biofilm ecosystem.

## Graphical abstract

Here, we describe the constitution of a model biofilm composed of four bacterial species, highly reproducible and robust against transient perturbations, in which we reveal a network of positive and negative interactions. Furthermore, we identified the molecular determinant of one negative interaction and demonstrated its importance for species distribution and its impact on biofilm robustness by mutational analysis of the multispecies ecosystem.

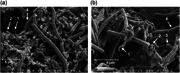

## 1 INTRODUCTION

In contrast to typical laboratory conditions of growth in liquid culture, bacteria in natural environments and those contaminating hospitals, or industrial and food‐processing procedures are more often found in multicellular surface‐associated communities known as biofilms (Costerton et al., 1987; Flemming et al., 2016; Hall‐Stoodley et al., 2004). Such biofilms are generally complex communities harboring numerous bacterial species in close spatial proximity (Elias & Banin, 2012). Diverse physical and social interactions between species take place in these communities. They are considered to determine not only the structure and spatial organization of the biofilm but also its global functions by modulating gene expression in the different species (Bridier et al., 2017; Burmølle et al., 2014; Liu et al., 2016; Rendueles & Ghigo, 2012). The physiology of each microbial species in complex, multispecies biofilms might be distinct from that in monospecific biofilms (L. B. S. Hansen et al., 2017; Liu et al., 2019). Moreover, multispecies biofilms exhibit emergent properties such as increased tolerance against antimicrobial agents (Bridier et al., 2012; Burmølle et al., 2006; Schwering et al., 2013; Yan & Bassler, 2019), synergistic degradation of toxic compounds (Breugelmans et al., 2008; Perera et al., 2019; Yoshida et al., 2009), stronger defense against protozoan grazing (Koh et al., 2012; Raghupathi et al., 2018), increased virulence in infection (Pastar et al., 2013; Wang et al., 2020) and protection against the action of biocides (Sanchez‐Vizuete, Le Coq, et al., 2015; Sanchez‐Vizuete, Orgaz, et al., 2015; Yan & Bassler, 2019). Studies on multispecies biofilms have also reported enhanced stress resistance, productivity, or biomass production (Burmølle et al., 2006; Lee et al., 2014; Liu et al., 2019; Ren et al., 2015), and, importantly, “community‐intrinsic properties” (Madsen et al., 2018) emerging from the social interactions between members of the biofilm and which may be important for its interaction with its environment.

It is thus important to decipher these interactions, positive or negative, at the molecular and biochemical levels to better understand the ecological and evolutionary factors that drive community function in natural or engineered systems (Rice et al., 2016; Ziesack et al., 2019). However, the number and types of interactions within multispecies biofilms are expected to grow very rapidly with the number of species present in the biofilm (Røder et al., 2016). Characterization of the interactions in complex biofilms and their underlying molecular mechanisms remains a challenge, as well as the evaluation of the importance of these interactions for the overall robustness of the structure and functionalities of these biofilms (Røder et al., 2020).

Here, we constructed a biofilm community from four species isolated from a biofilm consortium contaminating a milk processing plant, to analyze interspecies interactions and robustness to environmental stresses. This multispecies biofilm was highly reproducible, allowing us to use it as a model to study the dynamic interactions that take place between the species during its development. We were thus able to test the resistance of this complex biofilm and its formation process towards different continuous or transient perturbations. Then we identified the molecular determinant and assessed the contribution of one major interspecies interaction to the overall robustness of the biofilm.

## 2 MATERIALS AND METHODS

### 2.1 Bacterial strains and culture conditions

*Bacillus cereus* ATCC14579^T^ and *B. cereus* ATCC10987 were obtained from American Type Culture Collection (ATCC; https://www.lgcstandards-atcc.org). The 13 other bacterial strains used in this study (following paragraph and Table 1) were isolated from a biofilm formed on a gasket in a milk pasteurization line (Mettler & Carpentier, 1997). Strains CLL49 and CCL56 were identified respectively as *Pseudomonas fluorescens* and *Kocuria varians* by 16S ribosomal RNA gene sequence analysis. Strain CCL5 could be identified to the genus level as *Rhodocyclus* sp. The strains were routinely cultivated at 30°C on tryptic soy agar (TSA) plates (Becton Dickinson) or in liquid tryptic soy broth (TSB; Becton Dickinson) with agitation at 60 rpm.

**Table 1:** Screening of a collection of bacterial strains for mono‐ and five‐species biofilm formation

	Single‐species biofilm formation	Persistence in five‐species biofilm
*Bacillus* sp. CCL9	+	
*Bacillus cereus* ATCC14579^T^	+	+
***Staphylococcus hominis* CCL2**	+	−
*Staphylococcus hominis* CCL44	+/−	nd^a^
***Staphylococcus hominis* CLL45**	+	−
*Staphylococcus capitis* CCL1	+	nd
***Staphylococcus capitis* CCL15**	++	−
*Staphylococcus epidermidis* CCL10	+	nd
***Staphylococcus epidermidis* CCL75**	+	−
*Kocuria varians* CCL54	+	nd
***Kocuria varians* CCL56**	+	+
***Kocuria varians* CCL73**	+	−
*Rhodocyclus sp* CCL5	++	+
*Pseudomonas fluorescens* CCL49	++	+

^a^ Not done (experiment not performed).

### 2.2 Screening of bacterial strains isolated from a milk pasteurization line

Ability to form biofilms was assessed using a previously described microtiter plate method (O'Toole et al., 1999). To choose the most suitable strains for inclusion in the model system, the screening process was carried out in two stages. First, the *Staphylococcus* and *Kocuria* isolates were tested for their ability to form mono‐species and two‐species biofilms (The selected strains, three *Staphylococcus* and two *Kocuria*, are indicated in bold in Table 1). Their persistence in a five‐species biofilm was then assessed using a consortium containing *B. cereus* ATCC14579^T^, *Rhodocyclus* sp. CCL5, *P. fluorescens* CCL49, together with one each of the selected *Staphylococcus* and *Kocuria* strains (six different combinations were tested).

### 2.3 Experimental model biofilm formation

The biofilm development model of Maris (Maris, 1992) was adapted to the consortium of *Rhodocyclus* sp., *P. fluorescens, K. varians*, and *B. cereus* on submerged stainless steel chips (AISI 304; 12 × 25 × 1 mm; Goodfellow). Before use, they were prepared as previously described (Leriche & Carpentier, 1995). Cultures in the mid‐exponential growth phase were diluted into a fresh TSB medium to give a mixture containing 5 × 10^6^ cfu ml^−1^ of each species. A sterile stainless steel chip was covered with 300 µl of this mixture and incubated at 30°C in a humid atmosphere for 90 min to allow bacterial adhesion. Nonadherent cells were removed by gentle rinsing with 20 ml of TSB diluted to 5% in water (1/20 TSB). For biofilm formation, inoculated chips were incubated at 30°C in polystyrene Petri dishes, 9‐cm diameter, containing 20 ml of 1/20 TSB. The growth medium was changed every 24 h. For bacterial enumeration, the chips were rinsed with 1/20 TSB, then transferred to sterile pots containing 10 ml of 0.9% NaCl. Bacteria forming the biofilm were suspended by scraping with a sterile loop and disruption by ultrasonication (Branson 5200) for 3 min. Suspensions were diluted serially and plated in duplicate on selective agar plates: TSA supplemented with 8 mg L^−1^ chloramphenicol for the growth of *P. fluorescens* alone, TSA supplemented with 20 mg L^−1^ kanamycin for the growth of *Rhodocyclus* sp. alone, TSA supplemented with 15 mg L^−1^ oxolinic acid for the growth of *K. varians* alone, or on nonselective TSA plates for *B. cereus*, which formed very large, distinctive colonies on this medium.

### 2.4 Planktonic coculture

Strains were individually precultivated in TSB at 30°C with agitation, harvested by centrifugation, and resuspended in 1/20 TSB. They were diluted and mixed as above to give 5 × 10^6^ cfu ml^−1^ of each strain in 20 ml 1/20 TSB. This planktonic coculture mixture was placed in a 9‐cm diameter Petri dish and incubated for 24, 48, or 72 h at 30°C with agitation at 60 rpm to prevent biofilm formation.

### 2.5 Determination of anti‐*Kocuria* activity

Planktonic cultures of *B. cereus* ATCC14579^T^ wild‐type and the corresponding thiocillin mutant (Δ*tclE‐H*; see below) strain were grown to stationary phase at 30°C in TSB. One milliliter of this culture was centrifuged, the supernatant retained, and the bacterial pellet resuspended in 20 µl of TSB. Volumes (5 µl) of the planktonic culture, of the bacterial pellet, and the supernatant were spotted onto TSA plates seeded with an overnight culture of *K. varians* CCL56, then incubated for 24 h at 30°C.

### 2.6 Mutant strain construction

The four structural thiocillin genes *tclE‐H* were deleted from the chromosome of *B. cereus* ATCC14579 as follows. Sequences flanking the *tclE‐H* region (812 bp upstream and 1132 bp downstream) were amplified by polymerase chain reaction (PCR) using oligonucleotide primers lant1 and lant2 (ACCGATC**GGATCC**AGGCCAACCGATCATTATCAC and CTTGAAAACCATGGACTCATCCCACCTACAAG) and lant3 and lant4 (TGGGTAGAGTCCATGGTTTTCAAGAAGCTTAATTGTTCTC and ACCGATC**AGATCT**GGGTATCACCAAAGCTAACG), respectively. The two fragments were joined by overlap PCR using lant1 and lant4, and the resulting product was digested with *Bam*HI and *Bgl*II and cloned into the vector pMAD (Arnaud et al., 2004). The resulting plasmid was used to transform the *B. cereus* ATCC14579^T^ wild‐type strain and generate the Δ*tclE‐H* mutant strain by allelic replacement as described.

### 2.7 Electron microscopy

Scanning electron microscopy (SEM) experiments were conducted as described (Couvigny et al., 2017). Images were acquired and analyzed at the Microscopie et Imagerie des Micro‐organismes, Animaux et Aliments (MIMA2) microscopy and imaging platform.

### 2.8 Estimation of growth parameters with a logistic model

To represent the growth of a single‐species population, we used a simple logistic equation. Adding a latency phase with a constant lag time = τ, the differential equation reads:

$$\frac{\mathrm{dx}}{\mathrm{dt}}\left(t\right)=\left\{\begin{array}{ll} 0 & \text{for}\;t<\tau ,\\ \mu \;x\left(t\right)\left(1-\frac{x(t)}{K}\right) & \text{for}\;t\geq \tau , \end{array}\right.$$

where t represents time (in hours h) and x(t) represents bacterial cell counts (in cfu). Besides the lag time τ (h), other modifiable parameters are maximal growth rate μ ($h^{-1}$), carrying capacity K (cfu) and initial bacterial counts $x_{0}=x(0)$. This equation generates classical S‐shaped dynamics, with population x(t) converging towards K (the carrying capacity). Integration of this differential equation leads to the well‐known logistic function:

$$x^{L}\left(t;x_{0},K,\mu ,\tau \right)=\left\{\begin{array}{cc} x_{0} & \text{for}\;t<\tau ,\\ \frac{Kx_{0}}{x_{0}+(K-x_{0})e^{-\mu (t-\tau )}} & \text{for}\;t\geq \tau . \end{array}\right.$$

Given an experimental growth curve $\left(T_{i},X_{i}\right)$, we set the initial condition $x_{0}$ to be the bacterial count immediately after the 90 min adhesion step and we estimated parameters K, μ, and τ to minimize the following (constrained) least‐square error:

$$\min_{K\geq 0,\mu \in \left[0,\mu_{\max}\right],\tau \in [0,T_{\max}]}\sum_{i}{\left[\log_{10}\left(x^{L}\left(T_{i};x_{0},K,\mu ,\tau \right)\right)-\log_{10}\left(X_{i}\right)\right]}^{2}.$$

The least‐square estimation was performed in Matlab (The MathWorks, Inc.), using the function lsqcurvefit (Optimization Toolbox), with bounds $\mu_{\max}=1\;h^{-1}$ and $T_{\max}=72\;h$.

### 2.9 Statistical analysis

Values for end‐point biofilm growth experiments are expressed as the mean ± standard deviation calculated from at least five independent experiments. For statistical analysis, cfu for each strain was expressed as logarithms, except where noted. Statistical significance of measured differences was determined using a two‐way analysis of variance with Tukey's posttest as implemented in “R”. For Figure 6, in which members were omitted from the biofilm community, the significance of changes in the growth of the species was estimated by the Mann–Whitney Wilcoxon test, compared to growth in a four‐species biofilm. In the particular case of biofilms growing in non‐diluted medium (Figures 4 and 8), cfu for *Rhodocyclus* were in all cases below the limit of detection, comparison with biofilms grown in other conditions was made by assigning random values between 10^2^ and 10^4^ cfu cm^−2^ (i.e., the detection limit plus or minus 1 on a log scale) to the *Rhodocyclus* measurements for each of the seven data sets obtained from biofilms grown in a non‐diluted medium. The procedure was repeated twenty times and the *p* values retained are the upper 95% confidence levels. No significant difference resulted in the estimated significance values when the range of random values assigned to the *Rhodocyclus* cfu was increased tenfold, nor when the values were reduced further.

## 3 RESULTS

### 3.1 Construction of a multispecies, model biofilm based on a natural ecosystem

To construct a model, multispecies biofilm we exploited an industrial biofilm consortium, isolated from an industrial food preparation device (Mettler & Carpentier, 1997), comprising 13 strains corresponding to 7 species belonging to 5 genera (Table 1). Based on their capacity to form monospecies biofilms under laboratory conditions, we chose the best biofilm formers from each genus to simplify the consortia and facilitate subsequent analysis (see Section 2 and Table 1). Among these selected strains, we substituted the *Bacillus* strain with a well‐studied and genetically tractable laboratory strain (*B. cereus* ATCC14579^T^). We then tested combinations of the strains, including a representative of each genus, for their ability to form a multispecies biofilm. None of the *Staphylococcus* strains persisted at measurable levels in the laboratory multispecies biofilms. Hence, the consortium selected for further analyses contained the following strains: *Rhodocyclus* sp. CCL5, *P. fluorescens* CCL49, *K. varians* CCL56, and *B. cereus* ATCC14579^T^ (henceforth referred to, for simplicity, as *Rhodocyclus, P. fluorescens, K. varians*, and *B. cereus*, respectively) thus representing four of the five genera initially present in the industrial biofilm.

### 3.2 Population dynamics during biofilm formation

**Figure 1:**
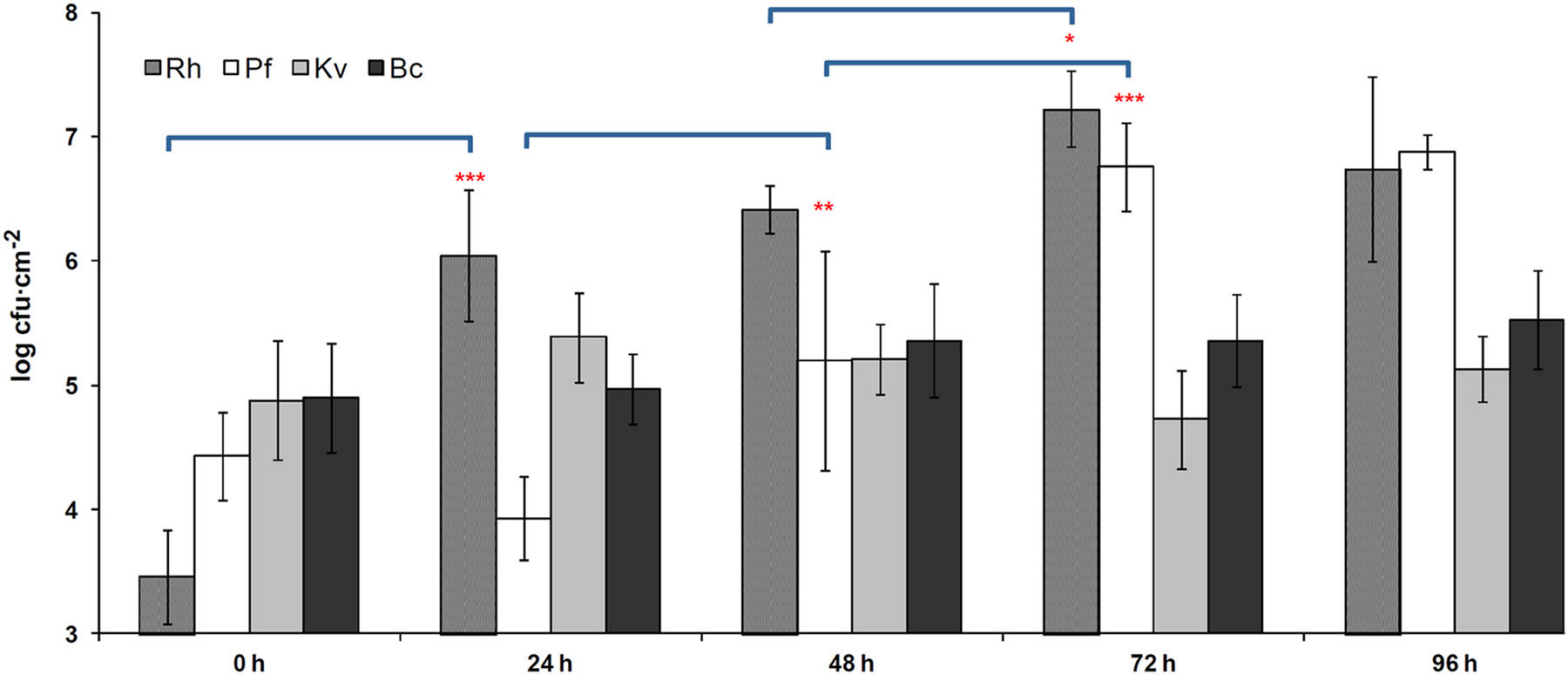
Evolution of species abundances during biofilm development. Times are in hours, after the 90‐min adhesion phase. Results are averages of at least five independent experiments; error bars correspond to one standard deviation. The evolution of the biofilm composition is analyzed in terms of changes of each species' abundance compared to that of the previous reading: *p < .05, **p < .01, ***p < .01 (Tukey's HSD). Bc, *B. cereus*; HSD, honestly significant difference; Kv, *K. varians*; Pf, *P. fluorescens*; Rh, *Rhodocyclus*

**Figure 2:**
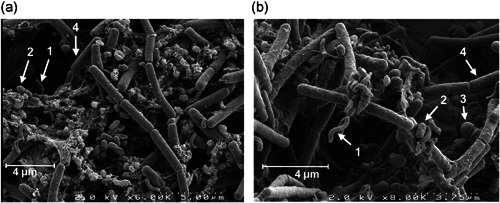
Physical Interactions between species in the biofilm. Scanning electron micrograph of the four‐species biofilm at *T* = 72 h (a) and of a biofilm grown under identical conditions but containing a *Bacillus cereus* Δ*tclE‐H* mutant lacking thiocillin production in place of the wild‐type strain (b). The four species are indicated by arrows in panels a and b: (1) *Rhodocyclus*, (2) *Pseudomonas fluorescens*, (3) *Kocuria varians*, (4) *B. cereus*. Note that in biofilms containing the wild‐type *B. cereus, K. varians* numbers are much reduced, such that none are observed in the image (a) which is representative of the majority of the observed microscopic fields. Scales are shown at the bottom right

We first characterized the global growth of the biofilm on stainless steel chips and the species dynamics during its development (Figure 1). Differential adhesion resulted in 2.9 × 10^3^ cfu cm^−2^ of *Rhodocyclus* at the starting time (*t* = 0 h) of the development phase of the biofilm, 2.7 × 10^4^ cfu cm^−2^ of *P. fluorescens*, 7.7 × 10^4^ cfu cm^−2^ of *K. varians*, and 7.9 × 10^4^ cfu cm^−2^ of *B. cereus*. This species composition changed considerably during the 4 days of biofilm development. *K. varians* and *B. cereus* populations stayed roughly constant throughout the entire experiment and became progressively subdominant compared to *Rhodocyclus* and *P. fluorescens*, which, together, dominated the community from 72 h onwards. While *Rhodocyclus* counts increased rapidly from the start of the experiment, the population of *P. fluorescens* remained constant at about 10^4^ cfu cm^−2^ for the first 24 h and increased thereafter. Populations of one or more of the four biofilm members varied significantly at each time point up until 72 h, whereafter cell numbers of each of the strains did not change significantly. Total population counts increased during the first 72 h of cultivation from 1.9 × 10^5^ to 2.3 × 10^7^ cfu cm^−2^ (though the growth kinetics of the different strains were notably dissimilar) and remained stable until the end of the experiment (1.4 × 10^7^ cfu cm^−2^ at 96 h). Hence, cell numbers and species distribution did not change significantly after 72 h, suggesting that the biological system has reached a steady‐state at Day 3, and 72 h was chosen as the endpoint in subsequent experiments. Observation of the 72‐hour old biofilm by SEM revealed a dense community with close contacts between cells. The only apparent structures consist of small aggregates of *P. fluorescens* cells and chains of *B. cereus* cells. In line with the small relative size of this population (comprising 1%–2% of the total cfu) and their small size, *K. varians* cells are hardly detectable (Figure 2a).

### 3.3 Community structure in biofilm versus planktonic lifestyles

**Figure 3:**
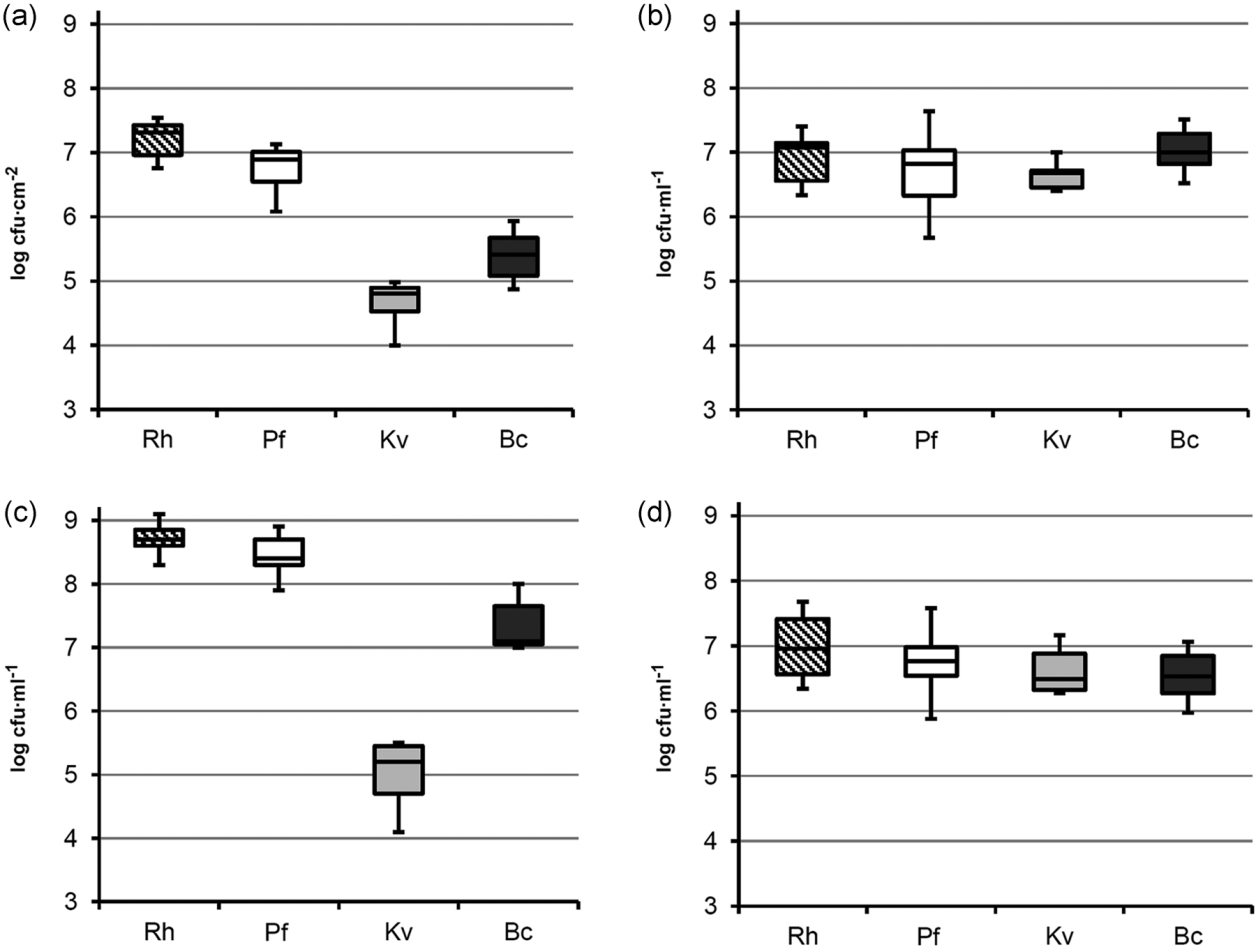
Effect of the mode of growth on community species distribution. (a) Species abundance in a four‐species biofilm after 72 h of cultivation; (b) abundance of each species in a planktonic coculture (72 h); (c) species abundance in the planktonic phase above the biofilms from which the data in (a) were determined. (d) as (b), wild‐type *B. cereus* being replaced by the Δ*tclE‐H* mutant. Boxes are defined between first and third quartiles with whiskers from minimum to maximum and median shown as a bold horizontal line. Data are derived from at least five independent experiments. Bc, *B. cereus*; Kv, *K. varians*; Rh, *Rhodocyclus*; Pf, *P. fluorescens*

To determine whether the steady state reached by the four‐species community is biofilm‐specific, we compared the population distributions in biofilm and liquid culture (Figure 3). The biofilm population structure (Figure 3a), with a majority of *Rhodocyclus* (73.6%) and *P. fluorescens* (25.2%) and considerably less of *K. varians* (0.2%) and *B. cereus* (1.0%) was markedly different from that of the planktonic grown community (Figure 3b). Proportions in liquid medium were more even, with *B. cereus, Rhodocyclus, P. fluorescens*, and *K. varians* comprising respectively 38.2%, 29.8%, 18.0%, and 14.0% of the total cell counts. Since surface‐associated biofilm populations release cells to the bulk liquid, and also recruit cells from the planktonic phase (Houry et al., 2012) it was interesting to determine whether the bacterial community composition present in the liquid phase above the biofilm was characterized by a biofilm‐like or by a planktonic‐like species distribution. As seen in Figure 3c, the composition of the biofilm supernatant community (*Rhodocyclus*, 62.6%; *P. fluorescens*, 34.6%; *B. cereus*, 2.8%; and *K. varians*, 0.01%) was more similar to that of the biofilm than to that observed in the pure planktonic culture. This suggests that, in the experimental setup, exchanges between biofilm and supernatant are more influenced by the seeding of the planktonic phase from the biofilm during the 24 h between washes than by the migration of cells from the planktonic phase to the biofilm. That is, the biofilm species composition is a property of the biofilm and not a reflection of the equilibrium attained in the planktonic phase. Thus, the equilibrium reached in biofilm appears specific to this mode of growth, and these results support the hypothesis that interspecies interactions are different in the biofilm compared to the planktonic culture, driving the system to a different equilibrium population distribution.

### 3.4 Robustness of the multispecies biofilm to perturbations

To evaluate the robustness of the biofilm, we applied transient or continuous changes of experimental conditions, and then compared the relative species abundances and the total cell numbers of the biofilm with those obtained under control standard conditions (see Section 2).

**Figure 4:**
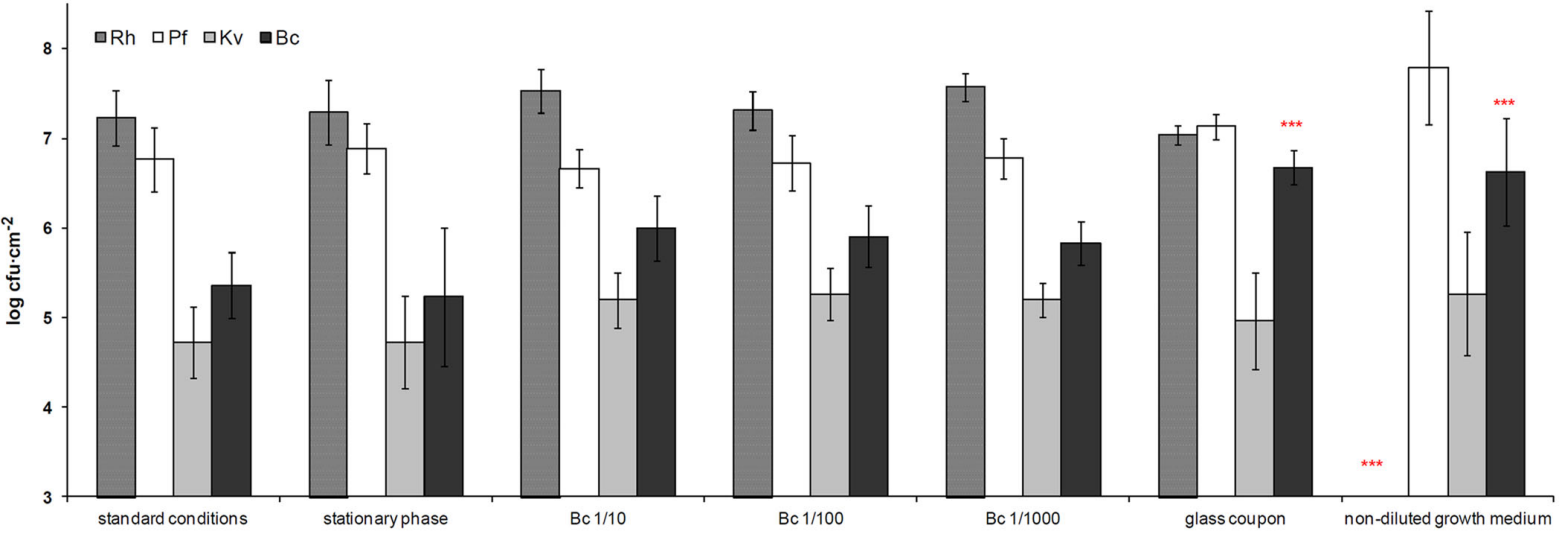
Effects of experimental perturbations on species distribution in the biofilm. Stationary phase: Inoculation with stationary‐phase cultures instead of exponential‐phase cultures; Bc 1/10, Bc 1/100, or Bc 1/1000: reduction of the amount of *B. cereus* in the initial inoculum from 5 × 10^6^ to 5 × 10^5^, to 5 × 10^4^, or 5 × 10^3^ cfu ml^−1^, respectively; glass coupon: replacement of stainless steel chip with glass; non‐diluted growth medium: growth in undiluted TSB instead of 1/20 TSB. Results are averages of five or more independent experiments; error bars correspond to one standard deviation. *p < .05, ***p < .001, significance (Tukey's HSD) of differences in species proportions relative to those in biofilms grown under standard conditions. Bc, *B. cereus*; HSD, honestly significant difference; Kv, *K. varians*; Pf, *P. fluorescens*; Rh, *Rhodocyclus*; TSB, tryptic soy broth

Neither total population counts nor species abundances at steady state were significantly altered by initial transient perturbations modifying either the global physiological state or the composition of the starting inoculum (Figure 4). In particular, drastic reduction in the inoculum (from 10^5^ to 10^2^ cfu ml^−1^) of *B. cereus*, the laboratory strain that has been substituted to the original *Bacillus* strain, did not change the relative species proportions and *B. cereus* was in all cases able to attain equilibrium cell densities (∼6.10^5^ cfu cm^−2^) demonstrating its capacity to grow in the biofilm. In contrast, two continuous perturbations did change the final structure of the community: Changes in the substrate surface properties (glass in place of steel) affected the equilibrium proportion of *B. cereus*, whose proportion increased from 1.0% to 16.3%, while the 20‐fold concentration of the culture medium affected both the biomass and species distribution of the biofilm (Figure 4). With this latter perturbation, total cell numbers increased threefold and relative species abundances were significantly modified. While *P. fluorescens* reached 93.3% of the total bacterial counts at 6.2 × 10^7^ cfu cm^−2^, *B. cereus* cell counts were increased 18‐fold to 4.3 × 10^6^ cfu cm^−2^ (6.5% of the total cell counts), *K. varians* remained a minority at 1.8 × 10^5^ cfu cm^−2^ (0.3%) and *Rhodocyclus*, the major component of the community under standard conditions, was no longer detectable (less than 1000 cfu cm^−2^). Less drastic modification of the culture medium (twofold concentration) had no measurable effect.

These results show that the establishment of the multispecies biofilm is resistant to initial, even quite considerable, transient perturbations, in contrast to its moderate response to change in the substratum and, especially, its marked response to major modification of the growth medium.

### 3.5 Dynamics of bacterial interactions during the formation of the four‐species biofilm

**Figure 5:**
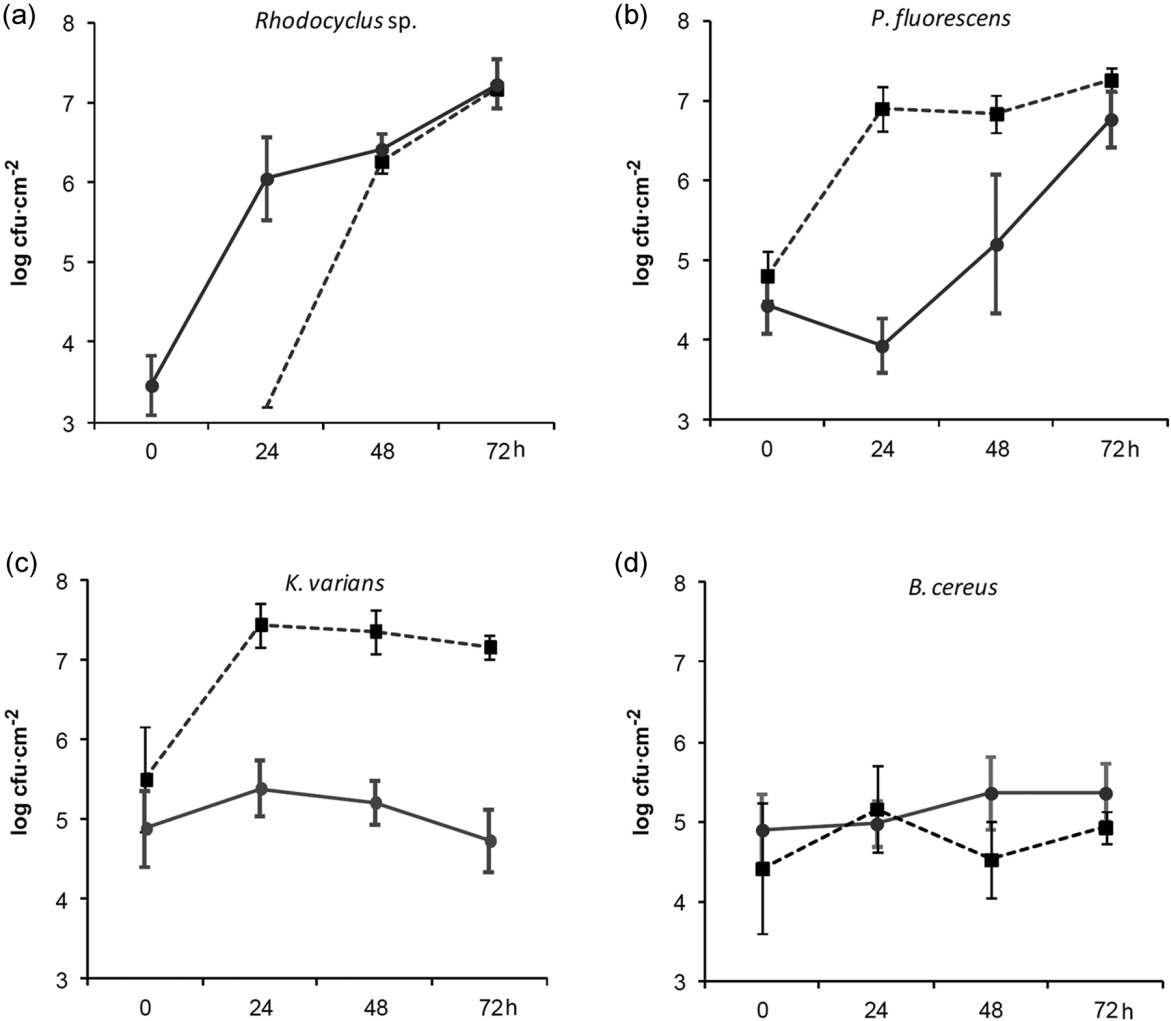
Bacterial growth in single‐species and four‐species biofilms. Cfu measured at 24‐h intervals in one‐species (dotted lines) and four‐species (solid lines) biofilms for (a) *Rhodocyclus*, (b) *Pseudomonas fluorescens*, (c) *Kocuria varians*, and (d) *Bacillus cereus*. Error bars correspond to one standard deviation; results are the averages of at least five independent experiments. cfu for *Rhodocyclus* growing as a single‐species biofilm were below the limits of detection at 0 and 24 h

To characterize the interspecies interactions that take place in the biofilm community, we compared population dynamics in pure and mixed biofilms. *P. fluorescens, K. varians*, and *B. cereus* adhered in similar numbers in monospecific or in four‐species experimental conditions (Figure 5b–d; 0 h time points). Total population counts in 72 h biofilms were also similar for three of the four species (*Rhodocyclus, P. fluorescens*, and *B. cereus*) in one‐species and four‐species biofilms. However, in the cases of *Rhodocyclus* (Figure 5a), *P. fluorescens* (Figure 5b), and *K. varians* (Figure 5c), the evolution of cell densities during biofilm formation differed greatly depending on the presence of the other species. *Rhodocyclus* cell density was considerably higher at early time points in the presence than in the absence of the other three strains. In contrast, *P. fluorescens* reached high cell density much more quickly when it was grown alone, and *K. varians* rapidly reached 200‐fold higher cell density in the monospecific biofilm than in the four‐species community, where its cell density remained approximately constant. Hence, with the possible exception of *B. cereus*, species interactions within the community have a considerable effect, either on the rate of increase towards equilibrium populations or on the maximum cell densities attained in the biofilm.

To gain insights into the mechanisms underlying these effects, we fitted a simple growth model to the experimental data and estimated growth parameters for the different species in single‐ and four‐species biofilms (Table 2). We used least‐square minimization to estimate three parameters: the maximal growth rate μ, the carrying capacity K and the lag time τ (see Section 2). For *Rhodocyclus* in a single‐species biofilm, the fit was not possible due to initial counts below the limits of detection (Figure 5a); while for *B. cereus* it was not relevant as the net growth rate within the biofilm is zero, both alone and in the biofilm community (Figure 5d). In the case of *K. varians*, the considerably reduced carrying capacity when present as part of a four‐species biofilm, as compared to its growth a single‐species biofilm, and its apparent lack of growth in the mixed community suggests that one or more of the other species have a negative influence on its growth. In the case of *P. fluorescens*, a negative effect is also observed, although of a different nature: parameter estimates indicated that growth rate and carrying capacity for *P. fluorescens* were comparable in the two cultivation conditions, whereas its lag time increased greatly when it was cultivated in the four‐species biofilm (Table 2). Since the adhesion step resulted in similar *P. fluorescens* populations at time zero (Figure 5b), it is apparent that *P. fluorescens* development was specifically delayed in the four‐species biofilm.

**Table 2:** Estimation of *K. varians* and *P. fluorescens* growth parameters in single‐ and four‐species biofilm using a logistic growth model

Organisms	Single‐species biofilm			Four‐species biofilm		
	*µ* (h^−1^)*	*K* (CFU/cm^2^)	τ (h)	*µ* (h^−1^)	*K* (CFU/cm^2^)	τ (h)
*Rhodocyclus* sp.	–	*1.5 × 10* ^7^	–	0.25	8.3 × 10^6^	0
*Pseudomonas fluorescens*	0.25	1.7 × 10^7^	0	0.94	5.8 × 10^6^	33
*Kocuria varians*	1.93	2.1 × 10^7^	3	–	*1.0 × 10* ^5^	–
*Bacillus cereus*	–	*7.6 × 10* ^4^	–	–	*2.3 × 10* ^5^	–

*Note*: The symbol “–” indicates that the estimation of *µ* and was inconclusive on the corresponding data set, either because of insufficient information at early time points (*Rhodocyclus* in single‐species biofilm) or because of an absence of apparent growth between *t* = 0 and 72 h (*K. varians* in four‐species biofilm and *B. cereus* in single‐ and four‐species biofilms), see Figure 4d. In those cases, the value indicated for the carrying capacity *K* is the mean value of the population in the corresponding data set.

Abbreviations: *µ*, growth rate; τ, lag time; *K*, carrying capacity.

In the case of *Rhodocyclus*, differences were seen in the initial development stages (Figure 5a). Adherent bacteria are undetectable in single‐species conditions (less than 1000 per square centimeter, compared with 3000 in the four‐species mixture), and remain undetectable after 24 h of growth. However, the population densities after 48 or 72 h are indistinguishable in the single‐species and four‐species mature biofilms. Thus, the presence of the three other species facilitates adhesion and initial development but does not increase the carrying capacity for *Rhodocyclus*.

Population densities of *B. cereus* at each biofilm development stage were similar in monospecies and four‐species biofilms (Figure 5d). Though the net growth rate of *B. cereus* in the biofilm under standard conditions is zero, this species is capable of multiplication within the biofilm since, when it is inoculated at a level of 2.5 × 10^3^ cfu cm^−2^, the final density nevertheless reaches 10^5^ (data for “Bc 1/1000” in Figure 4). Hence, *B. cereus* is neither favored nor disadvantaged in the multispecies biofilm.

These results reveal the existence of distinct types of interaction, both negative and positive between members of the multispecies biofilm. To further investigate these interactions, we assessed the effects of species omissions and substitutions on the final composition of the multispecies biofilm.

### 3.6 Effects of species omissions on biofilm composition

**Figure 6:**
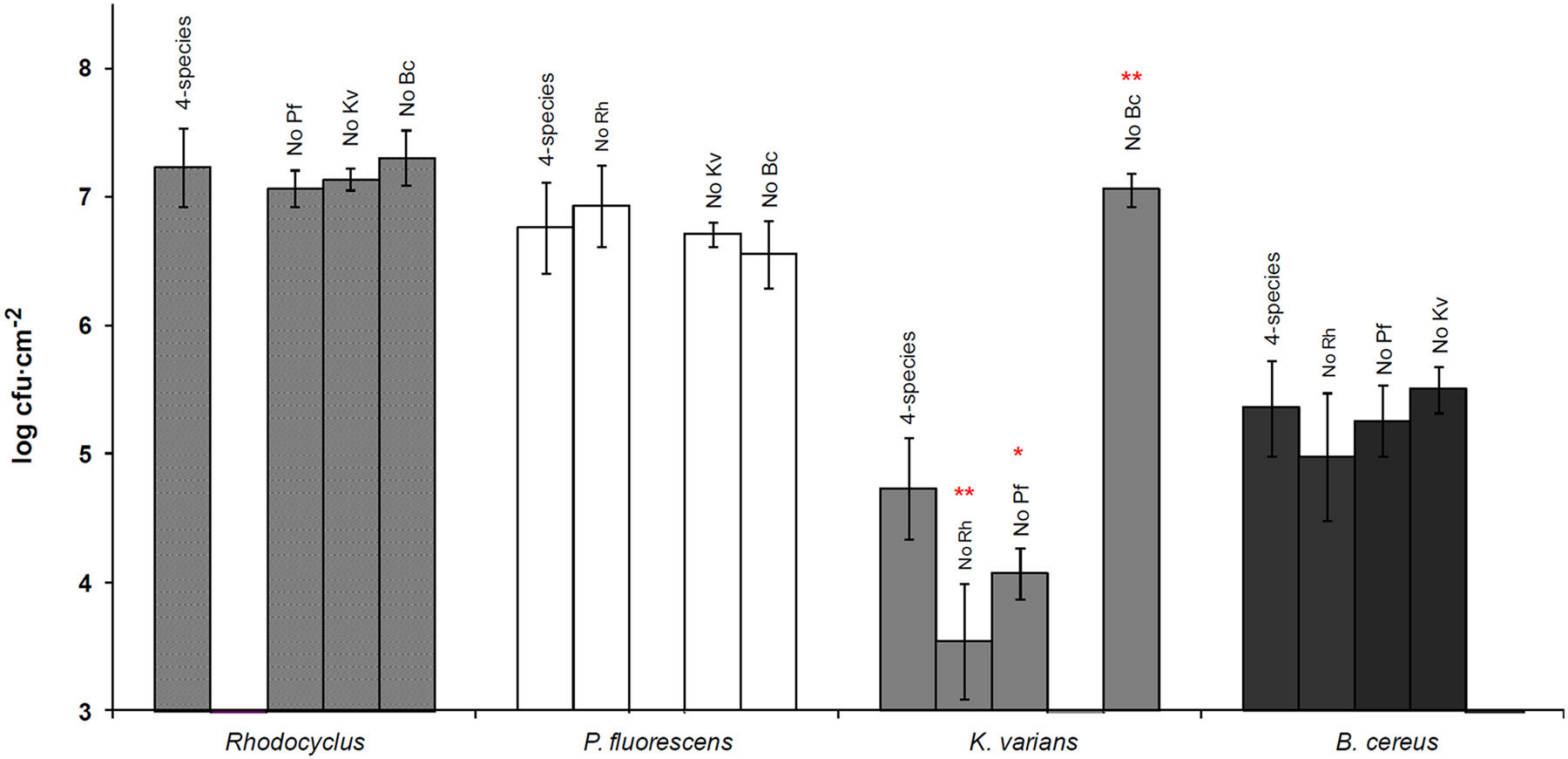
Effects of species omissions on biofilm composition. Bacterial counts were measured in biofilms grown under standard conditions for 72 h. Bars marked “4 species,” indicate bacterial counts in biofilms developed from inocula containing the four species in equal proportions; “no Bc,” “no Kv,” “no Pf,” and “no Rh” indicate the omission of *Bacillus cereus, Kocuria varians, Pseudomonas fluorescens*, or *Rhodocyclus*, respectively, from the initial inoculum. Error bars correspond to one standard deviation. Statistically significant differences, compared to “4 species” conditions, were estimated by the Mann–Whitney Wilcoxon test: *p ≤ .05, **p ≤ .01

We compared the community composition of each of the four possible three‐species biofilms with that of the four‐species biofilm. The abundances of *Rhodocyclus, P. fluorescens*, or *B. cereus* were not affected by the omission of any one of the other species, while, in contrast, the different omissions clearly affected the growth of *K. varians* (Figure 6). Specifically, the *K. varians* population was much greater when *B. cereus* was omitted from the community (1.1 × 10^7^ vs. 5.4 × 10^4^ cfu cm^−2^; *p* ≤ .01), and significantly lower in the absence of *P. fluorescens* (1.2 × 10^4^ cfu cm^−2^; *p* ≤ .05) or of *Rhodocyclus* (3.5 × 10^3^ cfu cm^−2^; *p* ≤ .01). These results demonstrate that *B. cereus* interacts negatively with *K. varians* and, since the growth of *K. varians* is inhibited while that of *B. cereus* is unaffected, the interaction is one of amensalism. Moreover, these results also show that the negative impact of *B. cereus* on *K. varians* is mitigated by the presence of *P. fluorescens* and *Rhodocyclus, K. varians* being less affected by *B. cereus* in the presence of these two other species than in their absence (compare the results for “4‐species” with “No Rh” and with “No Pf”).

### 3.7 Identification of the *B. cereus* product responsible for its negative interaction with *K. varians*

**Figure 7:**
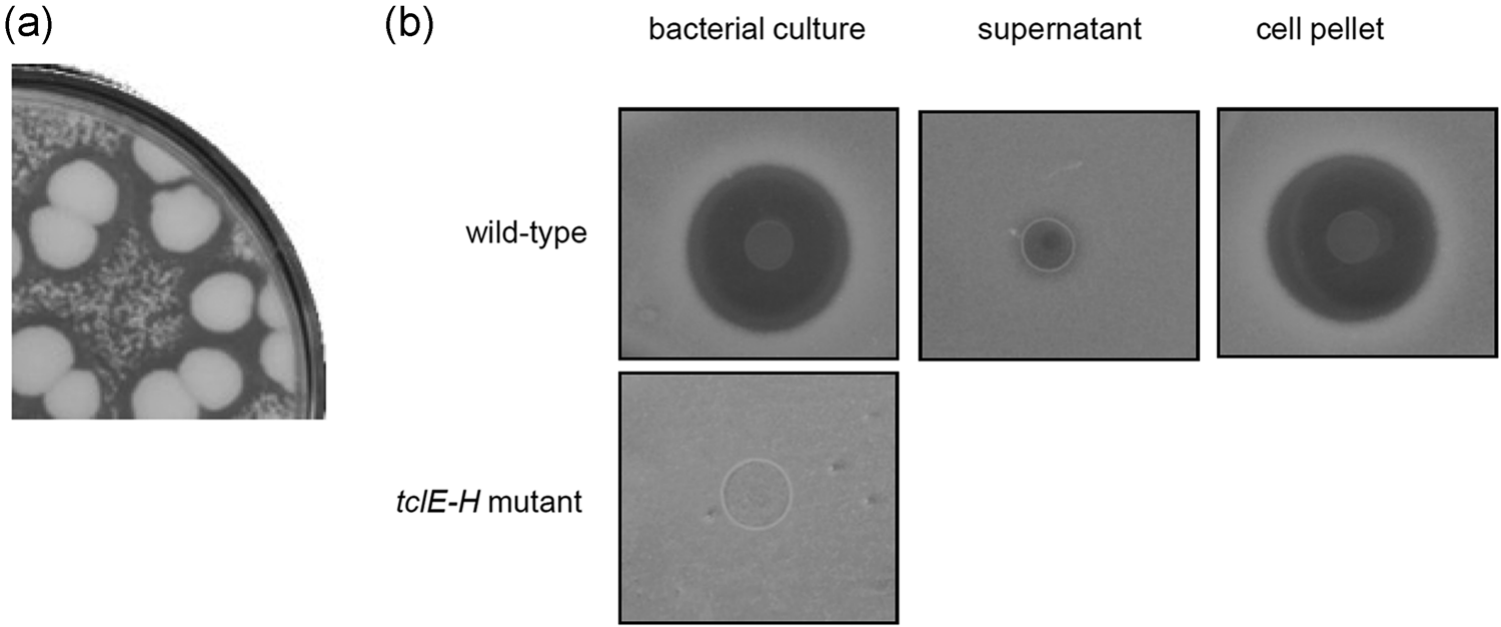
Detection of anti‐*Kocuria* activity. (a) Growth inhibition of *Kocuria varians* is seen around colonies of *Bacillus cereus* (large colonies) on nutrient agar plates. (b) A liquid culture of wild‐type *B. cereus* spotted onto a lawn *of K. varians* induces a zone of inhibition. The inhibitory activity remains associated with the cell pellet after separation by centrifugation of the liquid bacterial culture. The *B. cereus* Δ*tclE‐H* mutant has no inhibitory activity

**Figure A1:**
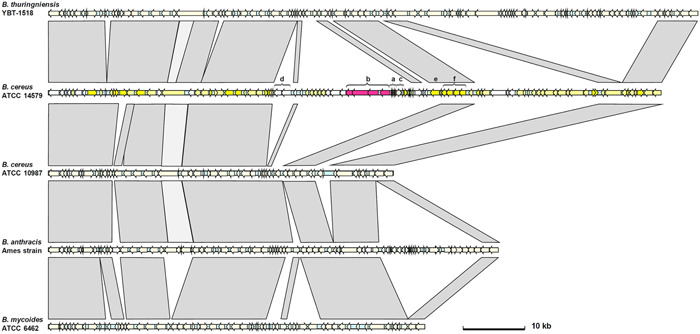
Alignments of genomic regions around the locus containing the thiocillin biosynthesis genes in *Bacillus cereus* ATCC14579. Genomes aligned are the two *B. cereus* strains used in this study ATCC14579^T^ and ATCC10987, and the representative genomes as defined at NCBI of other *B. cereus* group clades. Darker gray trapezoids join sequence blocks with over 90% base identity between strains; the lighter gray regions are at the position of a large and variable CDS (BC5055 in strain ATCC14579) containing peptidase and multiple mucin‐binding domains. CDS are colored light or dark yellow to distinguish transcriptional units as described by (Kristoffersen et al., 2012), Genome Biology, 13:R30); outside the annotated thiocillin production genes (red and pink), white indicates that no transcripts were detected in the latter study. Notable groups of genes in *B. cereus* ATCC14579 are marked: (a) thiocillin structural genes, four identical short ORFs, *BC5090* to *BC5087*, (b) lantibiotic synthesis enzyme genes, *BC5086* to *BC5083*, (c) polyketide resistance, *BC5091* and *BC5092*, (d) macrolide export, *BC5072* and *BC5071*, (e) cytolysin precursor *BC5101* (present in a number of *B. cereus* group strains, elsewhere on the chromosome of *B. anthracis* Ames, 70% predicted amino acid identity to perfringolysin O) and (f), outside the island, siderophore binding and import, *BC5106* to *BC5103*. The island in *B. anthracis* Ames is an integrated prophage

Colonies of *B. cereus* induced clear zones of inhibition of *K. varians* when these two strains were grown together on nutrient agar plates (Figure 7a), suggesting that this strain produces one or more inhibitory substances. Comparing the activities of different *B. cereus* strains, we noted that the strain ATCC10987 does not inhibit *K. varians* growth. Comparison of its published genome with that of the laboratory strain used in the present study, *B. cereus* ATCC14579, indicated a group of genes *BC5071* to *BC5102* which, except for one, were absent from ATCC10987. Further sequence comparisons confirmed that this region is highly variable from strain to strain in the *B. cereus–B. thuringiensis–B. anthracis* group (Figure A1). This region is part of the defined biosynthetic gene cluster *tclA‐X* (*BC5094* to *BC5071*) which is involved in the production of thiocillins, modified peptide antibiotics (Acker et al., 2009; Wieland Brown et al., 2009). Deletion of the four structural genes coding the thiocillin precursor, *tclE‐H* (*BC5090* to *BC5087*), produced a *B. cereus* mutant strain inactive against *K. varians* (Figure 7b). The anti‐*Kocuria* activity appears to remain mostly associated with the producing bacterial cells, or to be unstable, since very little is detected in the growth medium of a liquid culture of *B. cereus* (Figure 7b) and in addition, the community profiles of the four‐species planktonic cultures containing the wild‐type or the mutant *B. cereus* (see below) were similar (Figure 3, compare Panels b and d). In *B. cereus* the thiocillin I has a molecular mass of 1166 Da. Preliminary characterization of the *B. cereus* anti‐*Kocuria* activity indicated that it is sensitive to proteinase K and is associated with the 1 kDa fraction of dialyzed cell lysate, consistent with the properties described for thiocillin.

### 3.8 Elimination of the thiocillin‐mediated interaction between *B. cereus* and *K. varians* alters the biofilm composition and changes the response of the community to perturbations

**Figure 8:**
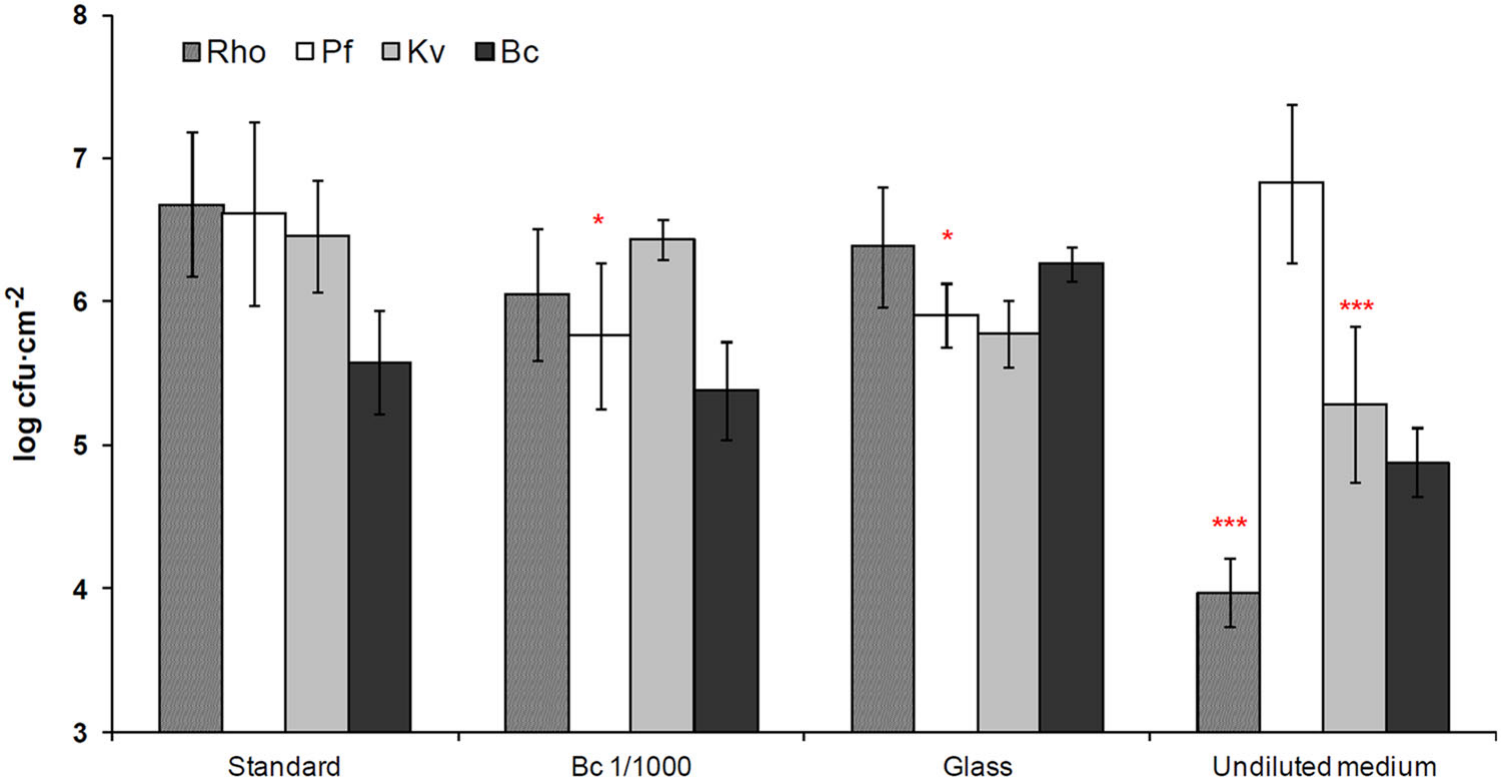
Effects of experimental disturbances on community species distribution of a four‐species biofilm developed with the *Bacillus cereus tclE‐H* deletion mutant strain. Biofilm composition was measured at 72 h post adhesion, data are represented as in Figure 1. Error bars correspond to one standard deviation, populations significantly different (Tukey's honestly significant difference test) from those measured in biofilms grown under standard conditions are noted: *p < .05, ***p < .001

The four‐species biofilm containing the *B. cereus tclE‐H* mutant in place of the wild‐type strain reached equilibrium at 72 h, like the wild‐type biofilm. However, its composition at 72 h differed from that of the standard wild‐type biofilm mostly in that, as may be expected, *K. varians* abundance was considerably higher (about 50‐fold; compare Figures 4 with 8). *Rhodocyclus* abundance was somewhat lower (around threefold), whereas *P. fluorescens* and *B. cereus* were little affected. Observation of this “mutant” biofilm by SEM revealed no apparent changes in the structure; however, in contrast with the standard biofilm, *K. varians* cells that form a much larger population became clearly visible (Figure 2b). We then investigated the importance of the thiocillin‐mediated negative interaction on the robustness of the system by submitting the “mutant” biofilm to transient or continuous perturbations as described previously. As seen in Figure 8, the growth of *P. fluorescens* was modified about sevenfold (*p* ≤ .05) by an initial transient perturbation (1/1000‐fold less *B. cereus* in the inoculum). The mutant biofilm was differently affected by continuous perturbations, relatively weakly when glass was substituted for the stainless steel surface (counts of *P. fluorescens* have decreased fivefold; *p* ≤ .05), and more considerably when the growth medium was concentrated 20‐fold, where counts of *K. varians* diminished approximately 15‐fold, and *Rhodocyclus* decreased by about 500‐fold (*p* ≤ .001 in both cases).

It can be seen that the responses of the mutant and wild‐type biofilms to continuous perturbations were different (compare Figures 4 and 8). Changing of the substratum material affected the *P. fluorescens* population in the mutant biofilm and that of *B. cereus* in the wild‐type biofilm. A more drastic modification was seen in the case of growth in the undiluted medium and was different for the biofilms containing wild‐type or bacteriocin‐mutant *B. cereus*. Both communities were dominated by *Pseudomonas*, with a minor or inexistent part of *Rhodocyclus*. However the more detailed species composition was clearly different: Whereas *Rhodocyclus* had been undetectable in the wild‐type biofilm grown in undiluted medium (i.e., cell counts were reduced by more than 10,000‐fold compared to those in biofilms grown under standard conditions), its numbers in the mutant biofilm remained at 10^4^ cfu cm^−2^ for growth in the undiluted medium. The undiluted medium resulted in a 20‐fold increase in *P. fluorescens* in the wild‐type biofilm but did not significantly affect its numbers in the mutant biofilm. *K. varian*s numbers were slightly increased (threefold, not statistically significant) by growth in the undiluted medium in the wild‐type biofilm but reduced 15‐fold in the mutant biofilm. These results indicate that interspecies interactions in the model biofilm are important in its reaction to perturbations.

Thus the two biofilms (with and without the effects of the *B. cereus* thiocillin) reached different, but stable equilibria under standard conditions. Neither is robust to strong continuous perturbations, and their respective reactions in terms of species compositions are distinct.

## 4 DISCUSSION

### 4.1 A model for multispecies biofilm with low complexity

Naturally occurring biofilms are multispecies ecosystems that constitute attractive opportunities to study interspecies interactions and community reactions to changes in their environment. Unfortunately, their complexity, combined with the difficulty of implementing controlled changes, limits their use in such studies. The model described in this study, developed from a selection of species from a biofilm isolated in a food industry setting, is of reduced complexity but demonstrates the positive and negative interactions characteristic of more complex biofilms. Because of its relative simplicity, it remains tractable in terms of biological analysis and mathematical modeling at the species level and can be used to provide answers to basic questions concerning the molecular mechanisms of interspecies interactions in microbial communities. The relative simplicity of the system may also play a part in its experimental reproducibility, this being a prerequisite for a laboratory model, while lack of reproducibility is a frequent problem in mixed biofilm studies (Røder et al., 2016). The quantity of biofilm and the relative proportions of each community member stabilized by 72 h (Figure 1). The reproducibility of the results (in particular the size of each bacterial population along time) and the approximate number of generations in each population during the experiment are incompatible with the hypothesis of mutations leading to more or less fitted subpopulations that would explain the changes in population size through time. Furthermore, one test of the biofilm dynamics under standard conditions performed using isolates from the 72 h biofilm of a previous experiment gave similar results as with stock isolates. Notably, cell counts of the minority species in the biofilm were stable as much as were the dominant species, demonstrating that they were not simply disadvantaged in the consortium, but attained an equilibrium, where positive and negative interactions, cell growth, and cell loss from the biofilm balanced each other.

### 4.2 Dynamic positive and negative interactions during growth of the biofilm

The final species distribution in the biofilm differs greatly from that at the initial stages of colonization: after the attachment phase, the biomass of two species, *P. fluorescens* and *Rhodocyclus*, strongly increased whereas that of the two other, *B. cereus* and *K. varians*, remained fairly constant (Figure 1). The three most abundant species (*P. fluorescens, Rhodocyclus*, and *B. cereus*) reached similar biomasses in one‐species and four‐species biofilms, ruling out the simple competition as the major force in defining the final species composition and indicating that species abundances were not limited by the carrying capacity of the substratum. The total cell numbers in the four‐species biofilm were higher than that of a biofilm composed of any one of the species alone, but they were not larger than the sum of those of the one‐species biofilms. As such, we do not see general interspecific cooperation in the mature, steady‐state biofilm, an observation in agreement with the results of Foster and Bell (2012) and in contrast to the conclusions of Ren et al. (2015). The absence of apparent general cooperation in the present biofilm could be related to its modest richness, with only four different bacterial species, and/or to the fact that these precise strains may not have cohabited for a long time: At the most 20 weeks (Mettler & Carpentier, 1997) for the three species isolated from an industrial device, as part of a larger community of microorganisms and under very different conditions from those of the experimental biofilm.

Except for *B. cereus*, the different species demonstrated notably different growth kinetics in four‐species biofilms compared to growth as single‐species, and each showed a different reaction to the growth in a mixed‐species community (Figure 5). *P. fluorescens* development was delayed in the four‐species biofilm while its final abundance was unchanged. The biofilm community interacted negatively with *K. varians* throughout its development and had a major impact on its final abundance, while it had a positive effect on *Rhodocyclus* at the attachment and initial growth phases. The striking effect in the four‐species biofilm on the adhesion and/or early growth of *Rhodocyclus* in the absence of changes in its final abundance (Figure 5a) suggests physical interactions aiding in attachment to the matrix, for example by modification of the substratum properties, or by epiphytic growth of one species on another. Growth of *B. cereus* was neither stimulated nor inhibited in four‐species biofilm, and maintained a constant, subdominant cell density in the biofilms throughout the experimental time frame, being able to grow to this level even when seeded at very low density (Figure 4). In addition, the final equilibrium populations at 72 h of the other species were not altered a 1000‐fold reduction of the *B. cereus* inoculum, suggesting that the equilibrium is maintained by interspecies interactions and is not a function of the history of the system, where, for example, space on the substratum might be irreversibly colonized by a species initially present as a high proportion of the total population.

It thus appears that the development and the final steady‐state of the biofilm is determined by a network of dynamic positive and negative interactions between the four species.

### 4.3 Molecular mechanism of interference competition by growth inhibition

We found that the negative effect on *K. varians* in the four‐species biofilm was due to the production of thiocillin by *B. cereus* and that this negative interaction was partly mitigated by the presence of *P. fluorescens* and *Rhodocyclus*. This bacteriocin is active against Gram‐positive bacteria, and *B. cereus* was found to inhibit the growth of all *Staphylococcus* tested, explaining the failure of these strains to form a mixed‐species biofilm (Table 1). The bactericidal activity of thiocillin has been reported to be associated mainly with the cell fraction (Wieland Brown et al., 2009), presumably because of thiocillin's poor solubility. Similarly, the inhibitory activity of *B. cereus* on *K. varians* was seen in conditions of growth at a close distance (on agar plates, Figure 7, or in biofilm, compare Figures 4 and 8), but not in a four‐species planktonic coculture (compare Figure 3b,d).

The coexistence of antagonistic strains in stable, or in cyclically evolving, proportions is predicted by theory for a range of interaction parameters (Chesson, 2000; Czárán et al., 2002; Hassell et al., 1994) and has been demonstrated in laboratory experiments (Kerr et al., 2002). Notably, these latter authors showed that antagonistic strains can coexist if interactions and dispersal occur at a local scale, coexistence under these conditions being associated with structured spatial organization of the strains. This biofilm structuration, which can protect sensitive species from the antagonistic effects of others (Kim et al., 2011), is also favored by positive interactions (Breugelmans et al., 2008; S. K. Hansen et al., 2007), such as the observed stimulation of *Rhodocyclus* adhesion by the biofilm community (Figure 5d). Nevertheless, the mechanism of mitigation by *P. fluorescens* and *Rhodocyclus* of the inhibitory action of *B. cereus* on *K. varians* may involve nonspecific protection by the physical presence of these two species—for example, physical separation and/or adsorption of the thiocillin molecule at the bacterial surface.

### 4.4 Interspecies interactions and robustness of the biofilm community

The establishment of the four‐species biofilm was resistant in the face of the transient perturbations that we tested, but the species composition changed in response to continuous perturbations, where the environment was permanently modified. We then looked more closely at the effect on biofilm community stability of one of the interspecies interactions, in particular, the negative effect of *B. cereus* on the growth of *K. varians*. As described above, the substitution of the *B. cereus* wild‐type by the thiocillin mutant strain altered the final composition of the biofilm, permitting increased growth of *K. varians* which became codominant together with *Rhodocyclus* and *P. fluorescens*. Neither the wild‐type nor the mutant biofilm was robust to strong continuous perturbations (compare Figures 4 with 8). Moreover, the mutant biofilm appeared to be less resistant than the wild‐type to transient perturbations, suggesting that in this model the major negative interaction may play a role in robustness that is only partially compensated by other stabilizing interactions (Burmølle et al., 2006; Lee et al., 2014). This is in agreement with the results of a study by Thompson et al. (2020) concerning a community composed of bacteria isolated from a potable water distribution system, where the authors detected redundant interspecies interaction effects. The interaction between one species and either one of two others had a positive effect on the biomass of their model biofilm, and this effect was more marked in the absence of a fourth, whose presence independently compensated for the loss of the interactions. Redundant interactions were also brought to light in our model biofilm but it must be remembered that the bacteria in the biofilm have had little time to coevolve together—probably 15–20 generations—and that the major negative interaction, though it may be important in this model, is not part of an evolved ecosystem.

Growth of the biofilm in the undiluted medium resulted in considerably higher numbers of *B. cereus* (Figure 4), possibly due to a capacity for exploiting the increased nutrient availability, to changed interactions in the biofilm community, or a combination of the two effects. In contrast, cell density of the *B. cereus* Δ*tclE‐H* was decreased in rich medium (Figure 8), both in relation to growth under standard conditions and to the wild‐type in the undiluted medium. These observations suggest that the differential reaction of the wild‐type and mutant biofilms to growth in the undiluted medium is at least partly explained by a modification of interspecies interactions other than competition.

## 5 CONCLUSIONS

Emergent properties of bacterial communities grown as biofilms, driven by social interactions, have huge implications for research and practical knowledge in such contexts as human health, food safety, rhizosphere role in plant growth, or even bioremediation. One approach to understanding these social interactions is to create and study artificial biofilm consortia in the laboratory. However, very few studies report such reconstructions of multispecies biofilm and elucidate the interspecies interaction networks that take place within. Moreover, the molecular determinant of these interactions and the analysis of their impact on the biofilm ecosystem properties have been reported in only a few studies. Here, we not only deciphered the active network of interactions that shapes a four‐species biofilm community and determine its robustness but also identified the molecular determinant of one of these interactions and revealed how it impacts the structure and properties of this community.

## CONFLICT OF INTERESTS

The authors declare that there are no conflict of interests.

## ETHICS STATEMENT

Ethics statement is not applicable to this study.

## AUTHOR CONTRIBUTIONS

**Aurélie Baliarda**: Conceptualization (equal), data curation (equal), investigation (equal), methodology (equal), validation (equal), writing – review & editing (equal). **Michèle Winkler**: Investigation (equal). **Laurent Tournier**: Conceptualization (equal), formal analysis (equal), methodology (equal), writing – review & editing (equal). **Colin Tinsley**: Conceptualization (equal), investigation (equal), methodology (equal), validation (equal), writing – original draft (equal), writing – review & editing (equal). **Stéphane Aymerich**: Conceptualization (equal), methodology (equal), project administration (equal), supervision (equal), validation (equal), writing – original draft (equal), writing – review & editing (equal).

## Data Availability

Raw data associated with this article are available at https://doi.org/10.15454/SNN1LB.
